# Neglected communities

**Published:** 2013

**Authors:** Hannah Faal

**Affiliations:** Chairperson: Africa Vision Research Institute, Durban, South Africa.

**Figure:**
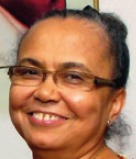
Hannah Faal

**The Gambia.** People conducting a population-based survey randomly selected the hamlet of Tunku. The survey team waded across flooded paddy fields, constantly asking for directions. Tunku turned out to be a cluster of five mud huts perched on a hill on the bank of the River Gambia. The team found two families and one blind old woman there. The villagers could only reach their homes from the river using dugout canoes, and they grew their crops in fields that flooded. Why would anyone choose to live here, in this ‘end of the road’ place?

***Nigeria.** Ibadan is an ancient city that grew around its traditional palace. Out of the city's overcrowded and labyrinthine streets, weeping mothers streamed daily to the tertiary teaching hospital less than half an hour away with children dying or dead from diseases such as malaria, diarrhoea, or marasmus. In the hospital, they saw clean wards with crisp white bed sheets* — *an unimaginable and unattainable luxury for them in their homes. Meanwhile, medical students ventured in the opposite direction, through the same labyrinthine depths and into homes in which gutters with filthy waste water ran through the middle of rooms and the latrine was just outside the window. Their purpose? A paediatric posting, but the challenges they found in these urban slums demanded a public health approach. Their traditional doctor's training was powerless in the face of such challenges.*

**What kinds of neglected communities are out there?**

- Those at the ‘end of the road’ in inaccessible areas
- Migrant communities
- Those in slums: pockets of poverty in urban areas
  Post-disaster communities
- Communities in areas of conflict or in refugee camps
- Communities affected by environmental degradation from mining, climate change or bad development projects.

‘NTDs affect people who are neglected in every facet of development’

**Nairobi, Kenya.** Known as a cantankerous old woman, she lived alone in a large house in the expensive part of town. The house and garden had seen better days. Someone came in to drop her daily meal and rushed out because of the strong smell of decay and the madness of the old woman. When she was found unable to walk because of a fall in her kitchen and forced to go to the clinic, it was discovered that she had blinding cataracts; her poor vision at least one of the causes of her apparent self-neglect. She epitomised neglect by self and by others – even in the absence of poverty.

Like the people in Tunku and in the slums around Ibadan, poor and/or remote communities around the world – whether rural or urban-are often neglected, whether in Africa, the Amazon, Australia or the Indian sub-continent. However, neglected people can even be found in affluent communities.

If we want to address neglected tropical diseases (NTDs), we have to focus on the communities affected. As David Molineux writes on pages 21-24, NTDs affect people who are neglected in a variety of ways: through poverty and lack of access to basic water and sanitation, health services, and education … In fact, just about every facet of development.

How can eye care programmes respond to the challenge of reaching these neglected people? Here are some examples of different strategies.

## Sri Lanka: into every home

Midwives, who function as family health workers, form the backbone of health service delivery in Sri Lanka. In 2000, there were over 5,000 midwives in the country, each responsible for between 3,000 and 4,000 people. This network should – in theory- reach the entire population of Sri Lanka. In rural Sri Lanka, it was noted at a Sightsavers review of primary eye care in 2010, that the midwives are welcomed into people's homes and know every household and family member intimately: the child whose Road to Health chart is kept by his literate mother (Sri Lanka has a 98% literacy rate), the grandfather with hypertension, and the grandmother reluctant to wear her glasses. One even helped a father who was determined to harvest rainwater, diga well, and build a latrine. By being so close to their local community, these health service workers are able to provide the right support, to the right person, at the right time.

**Figure:**
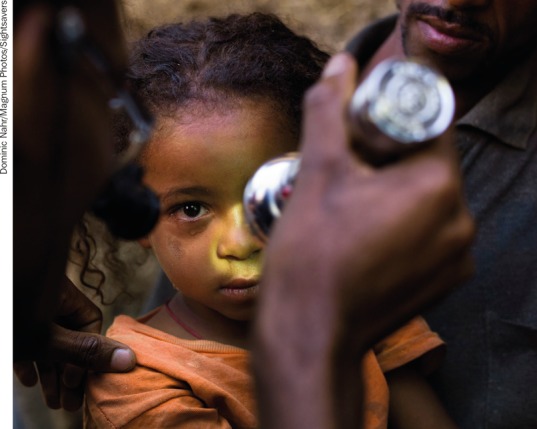
Genemo Abdela, health professional, is one of a team surveying almost 600,000 people in Ethiopia for the neglected tropical disease trachoma. ETHIOPIA

## Onchocerciasis in Africa

Onchocerciasis occurs among rural communities who live beside the fast-flowing rivers in which the black fly breeds. This fly is the ‘vector’ for transmission of onchocerciasis: the pathogen which causes the disease enters the bloodstream of people when they are bitten by the black fly.

Despite a major, decades-long programme of aerial spraying of the breeding sites, supplemented by treatment with the drug ivermectin, prevalence was still found to be high in some remote communities such as those in the highlands of Togo. A Special Intervention Zone was set up in Togo and in other countries with pockets of high prevalence in West Africa, and control measures were increased to ensure that people living in these remote areas were being protected.

Since its launch in 1995, the African Programme for Onchocerciasis Control (APOC) has developed community-directed strategies designed to ensure that even the most neglected communities could be reached. APOC defined the critical factors as being:

- commitment by everyone involved, from national to community level
- health system thinking: strengthening the health systems where they interact with the communities – on the ‘front
  line’ of health care delivery
- paying attention to broader issues: gender and equity, health workers’ roles, and community development and awareness
- encouraging the community in its role to recruit and support the workers
- engaging and empowering the communities in the partnership.

Adoptionof these strategies by governments has led to successful control of onchocerciasis in Africa, so that in many areas there is the real possibility of elimination of the disease. In addition, these strategies have formed the basis for control of other neglected tropical diseases, like trachoma, and have been adapted to address other health and development programmes meeting the needs of some of the poorest and most neglected people.

## Ghana: reaching remote island communities

In the 1950s, the leader of Ghana, Kwame Nkrumah, had anticipated his country's electricity needs and built a dam across the Volta river. As the surrounding countryside became flooded, hilltops became islands. Yet communities clung to these islands, and illiterate mothers kept their children's Road to Health charts beautifully wrapped in plastic, most of the cells not filled as the immunisation dates were missed.

In response, the health administration, with World Health Organization (WHO) support, focused their attention on the Volta region with the ‘Reaching Every District’ (RED) approach. Selected districts were provided with funds and technical assistance to develop and implement micro-plans that covered outreach services, supervision and monitoring, social mobilisation, quarterly review meetings and other community activities in each of these remote communities. In addition, some districts received motorbikes and outboard motor boats to help them reach every island community and provide basic health services, including immunisation, vitamin A supplementation, de-worming medicines, and disease surveillance.

## Where to now?

At the global level, policies emphasise the need to reach the poorest. Examples include Millennium Development Goal 1: the reduction of poverty and hunger, and the UN Human Development Index, which measures health, education and living standards. The WHO has addressed neglect of populations and communities by focusing on social determinants of health, equality issues, universal coverage, a human rights approach to health and a return to the primary health care philosophy. Advocacy has achieved a global determination to eliminate neglected diseases.

## What can an eye care worker do?

The first step towards bringing better health and eye health to neglected communities, is for eye care workers to familiarise themselves with national strategies for reaching neglected communities. The second step is to create a map of the area covered by the district eye health programme (using criteria for neglect which have been defined by the health services). This will help to identify hard-to-reach or particularly disadvantaged communities that must be prioritised.

Eye health workers should look beyond just the eyes', and aim to be part of a campaign to take health into every home throughout the year. The trainers of eye health workers should ensure that they teach eye health workers about neglect in all its facets, its causes and its solutions, and the need to engage with other agencies working in health.
